# Mesoporous Polymeric Ionic Liquid via Confined Polymerization for Laccase Immobilization towards Efficient Degradation of Phenolic Pollutants

**DOI:** 10.3390/molecules28062569

**Published:** 2023-03-12

**Authors:** Yu Liang, Xinyan Chen, Jianli Zeng, Junqing Ye, Bin He, Wenjin Li, Jian Sun

**Affiliations:** 1 Key Laboratory of Molecular Medicine and Biotherapy, Ministry of Industry and Information Technology, School of Life Science, Beijing Institute of Technology, Beijing 100081, China; 2 State Key Laboratory of Catalytic Materials and Reaction Engineering, Research Institute of Petroleum Processing, China Petroleum & Chemical Corporation, Beijing 100083, China; 3 Advanced Research Institute of Multidisciplinary Science, Beijing Institute of Technology, Beijing 100081, China

**Keywords:** polymeric ionic liquids, confined polymerization, laccase, immobilization, 2,4-DCP, removal

## Abstract

Laccase immobilization is a promising method that can be used for the recyclable treatment of refractory phenolic pollutants (e.g., chlorophenols) under mild conditions, but the method is still hindered by the trade-off limits of supports in terms of their high specific surface area and rich functional groups. Herein, confined polymerization was applied to create abundant amino-functionalized polymeric ionic liquids (PILs) featuring a highly specific surface area and mesoporous structure for chemically immobilizing laccase. Benefiting from this strategy, the specific surface area of the as-synthesized PILs was significantly increased by 60-fold, from 5 to 302 m^2^/g. Further, a maximum activity recovery of 82% towards laccase was recorded. The tolerance and circulation of the immobilized laccase under harsh operating conditions were significantly improved, and the immobilized laccase retained more than 84% of its initial activity after 15 days. After 10 cycles, the immobilized laccase was still able to maintain 80% of its activity. Compared with the free laccase, the immobilized laccase exhibited enhanced stability in the biodegradation of 2,4-dichlorophenol (2,4-DCP), recording around 80% (seven cycles) efficiency. It is proposed that the synergistic effect between PILs and laccase plays an important role in the enhancement of stability and activity in phenolic pollutant degradation. This work provides a strategy for the development of synthetic methods for PILs and the improvement of immobilized laccase stability.

## 1. Introduction

Chlorophenols, a class of important chemicals that are used in the industrial field, can cause serious pollution due to their aromatic structure and high toxicity [1,2]. Therefore, the degradation of chlorophenol pollution is urgent and has attracted extensive attention. Although a variety of physical, chemical, and biological methods have been used towards this end, most of these have certain limitations, such as high energy consumption, high costs, and low degradation efficiency [3,4,5], limiting their large-scale application. In recent years, biodegradation has emerged as one of the most promising technologies for treating harmful organic pollutants from the viewpoints of sustainable chemistry and green chemistry, due to its higher environmental friendliness, higher selectivity, and more efficient mineralization compared with other methods [6]. Further, the potential for biodegradation to be used to remove chlorophenols in water has attracted considerable research attention.

Laccases (EC 1.10.3.2), found in fungi, plants, bacteria, and insects, belong to the blue-copper enzyme family. They have broad substrate specificity and eco-friendliness [7]. In addition, laccases can catalyze the one-electron oxidation of various organic and inorganic substrates, including phenols, ketones, phosphates, ascorbates, amines, and lignin. Free laccases extracted from nature have the disadvantages of poor stability, a short lifespan, being difficult to recycle, and high cost, meaning it is generally difficult for them to tolerate harsh industrial processing conditions [8,9,10]. Researchers have mainly modified enzymes through protein engineering, non-covalent modification, chemical modification, and immobilization [11,12,13]. However, in practice, protein engineering still faces many challenges. After protein modification, laccases exhibit unstable properties with regard to their preservation and application [14]. Both the covalent modification and non-covalent modification of enzyme systems are unstable, and the activity and stability of enzymes may be reduced after modification. Therefore, enzyme immobilization technology is ideal for compensating for the deficiency of free enzymes [15,16]. One of the significant advantages of enzyme immobilization is that it provides an expected ideal enzyme system that can be reused for a couple of cycles, reducing operation costs [11,17]. Currently, enzyme immobilization strategies mainly focus on supports innovation through the use of metallic compounds [18], carbon-based materials [19], silicon-based nanomaterials [20], metal-organic frameworks or their derivatives [14], polymers [21], etc. Liu and co-workers immobilized laccase into carbon-based mesoporous magnetic composites through adsorption. The system was able to retain above 70% of its initial activity after five cycles [22]. Navarro-Sanchez and colleagues encapsulated laccase in MOF and realized high activity of laccase under high-temperature conditions [23]. Xu and colleagues developed a method of laccase immobilization based on nanofibrous membranes consisting of electrospun chitosan/poly (vinyl alcohol) composites. Compared with free laccase, the combined removal of 2,4-DCP was significantly improved by the immobilized laccase [21]. However, there are still some challenges that need to be resolved prior to enzyme immobilization’s industrial application, such as enzyme leakage, toxicity, the low surface area involved, and the supports’ high cost. Therefore, it is highly desirable to develop novel supports to achieve the effects of the high load, low leakage, high activity, and high stability of enzymes.

In the past few decades, the application of polymeric ionic liquid (PIL)-based biomaterials has attracted wide attention. PILs are one type of conductive polymer that have an IL structure in the repeating unit, where the anions or cations of the PILs are confined in the matrix of the macromolecules. They have a wide range of applications in polyelectrolytes, flexible materials, stimuli-responsive materials, catalysts, energy materials, and carbon materials [24,25,26,27]. To the best of our knowledge, the application of PILs in the immobilization of enzymes is still under development. There are two factors limiting this application. On the one hand, the existing preparation methods for PIL-based porous materials involve low IL contents and a narrow application range [28,29]. On the other hand, although PILs can have high IL contents, they are mostly non-porous or low-specific surface area materials [24,28,29]. Recently, Wang and colleagues introduced hydrophilic polyvinylpyrrolidone chain segments into hydrophobic polydivinylbenzene frameworks through the reinitiation of suspended double bonds, affording a kind of adsorbent with a uniform distribution of functional groups, strong cross-linking structure, and highly specific surface area [30]. Therefore, a higher specific surface area of PILs can be achieved through this kind of confined polymerization, further broadening their application in the biological field. However, research into this subject is still lacking. The higher the specific surface area, the better the enzyme immobilization’s performance, owing to there being more available reactive groups [31,32]. Therefore, the strategy of enhancing the specific surface area and mechanical properties of PILs is expected to further broaden their application in the biological field [33,34].

Herein, two amino-functionalized PIL microspheres synthesized by traditional free radical polymerization and confined polymerization, respectively, were compared. They were found to have different specific surface areas, pore structures, and rough surfaces. Laccase was covalently immobilized on the surface of these microspheres via the bridging of glutaraldehyde. A better enzyme immobilization system was selected for the degradation of 2,4-dichlorophenol (2,4-DCP), and its biocatalytic degradation was characterized. The optimum conditions, including the temperature and pH, for the immobilized laccase system were determined using the enzyme relative activity test, and the storage stability and circulation of the same system were further tested. Finally, the biodegradation of 2,4-DCP was systematically evaluated. This work provides a protocol for the design and optimization of the structure of PILs. In addition, the study found that compared with free laccase, the stability and circularity of the PIL-immobilized laccase were greatly improved.

## 2. Results and Discussion

### 2.1. Structural Characterization of Polymerized Ionic Liquid Materials

**Scheme 1:**
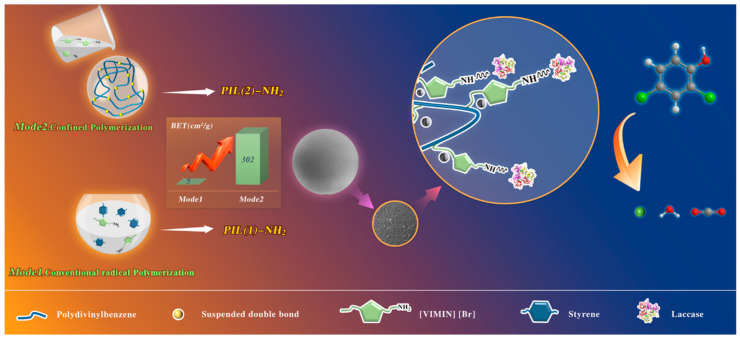
Preparation of PILs for laccase immobilization and its application in phenol removal.

As illustrated in Scheme 1, the amino-functionalized PILs were synthesized by two different polymerization methods (i.e., traditional free radical polymerization and confined polymerization), and the corresponding products were denoted as PIL (1)–NH_2_ and PIL (2)–NH_2_, respectively. The material synthesized after the immobilization of laccase was recorded as PIL–NH_2_–GA–Lac.

**Figure 1:**
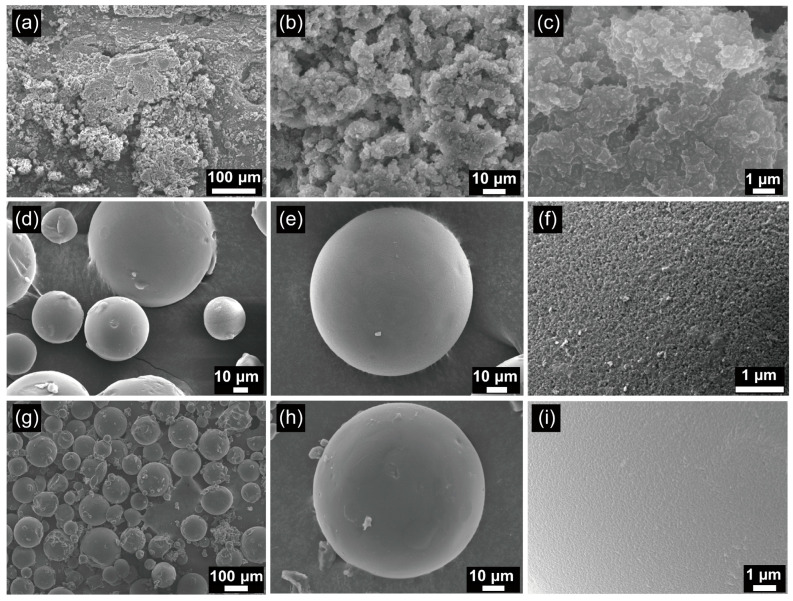
SEM micrographs of PIL (1)–NH_2_–GA–Lac (**a**–**c**), PIL (2)–NH_2_ (**d**–**f**) and PIL (2)–NH_2_–GA–Lac (**g**–**i**).

Figure 1a–c show the morphology of the PIL–NH_2_–GA–Lac composites. The PIL (1)–NH_2_ composites did not have regular spheroids, nor were they observed to have a uniform surface or pore structure. Figure 1d–f indicate that PIL (2)–NH_2_ had a spherical structure with a size distribution between 15 and 100 μm, and the surface of its microsphere was rough, as shown in Figure 1f. In addition, PIL (2)–NH_2_–GA–Lac (Figure 1g–i) still retained a spherical structure, while its surface was smoother compared with that of the pristine support (PIL (2)–NH_2_). This may have been due to the coating that formed after the immobilization of the laccase.

**Figure 2:**
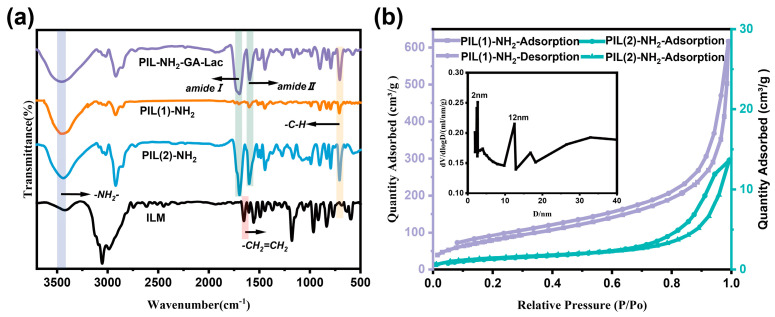
FTIR spectra of PIL–NH_2_ and PIL–NH_2_–GA–Lac (**a**). Nitrogen adsorption–desorption isotherms and BJH pore size distributions of PIL–NH_2_ (**b**).

Surface interactions between the laccase and two supports (i.e., PIL (1)–NH_2_ and PIL (2)–NH_2_) were investigated by FT-IR (Figure 2a). As expected, in the spectra of the IL monomer and PIL–NH_2_, a stretching vibration peak of the N-H bond of a primary amine was observed near 3441 cm^−1^, and a vibration peak near 1182 cm^−1^ also confirmed the presence of a primary amine. The absorption corresponding to the C-H stretching of the -CH=CH of the ILM was observed at 3026 cm^−1^, which indicated the aromatic structure of DVB. The peak at 1652 cm^−1^ corresponded to the characteristic peak of the carbon–carbon double bonds of -CH=CH_2_, and the peak at 709 cm^−1^ was the C-H characteristic peak in the double bond connecting the benzene ring, which was not affected by the introduction of functional groups [35]. This also verified the successful synthesis of the polymer skeleton. Moreover, the peaks at 1691 cm^−1^ and 1602 cm^−1^ represented the presence of an amide I and an amide II band, respectively, which indicated that laccase was immobilized to the amino-functionalized PIL [36].

Figure 2b shows the specific surface area analysis and pore size distribution curves of the two amino-functionalized PIL supports. It can be observed that the adsorption curve of the isotherm was inconsistent with the desorption curve, resulting in hysteresis loops. The N_2_ isothermal adsorption–desorption curves of PIL (1)–NH_2_ and PIL (2)–NH_2_ were IV H3 and H4 hysteresis loops, respectively. It was found that PIL (1)–NH_2_ had a fairly low specific surface area of 5 cm^2^/g. Nevertheless, PIL (2)–NH_2_ showed a high uptake capacity, having a Brunauer–Emmett–Teller (BET) surface area of 302 cm^2^/g. The formation of a high specific surface area can be attributed to the polymerization of the polystyrene skeleton in the early stage of the procedure [30]. The analysis of the pore size distribution in PIL (2)–NH_2_ showed that it had a mesoporous structure with a pore size of mainly 12 nm. Its pore size distribution between 12 and 50 nm can be interpreted as owing to the diversity of its pore patterns. According to the previous literature, a part of laccase can be immobilized into mesoporous materials, further improving its immobilization efficiency and activity [23,37].

To verify the effect of the specific surface area on the immobilized efficiency, we used the popular crosslinking agent glutaraldehyde (GA), and the amount used (2% *v*/*v*) in this work was analogous to that used in other studies (0.5–5% *v*/*v*) [26,38]. Glutaraldehyde is a crosslinking agent that has two aldehyde groups during the cross-linking process. In this study, after activation, an aldehyde group was covalently linked to PIL–NH_2_ and a free formyl of the microspheres. During the immobilization of laccase, free formyl groups on the surface of PIL–NH_2_ microspheres were covalently linked to laccase macromolecules. However, excessive cross-linking can lead to distortion of the laccase configuration, so a relatively low concentration of glutaraldehyde was used for immobilization to prevent the distortion of the enzyme structure and to reduce the activity [39].

### 2.2. Immobilization of Laccase

**Figure 3:**
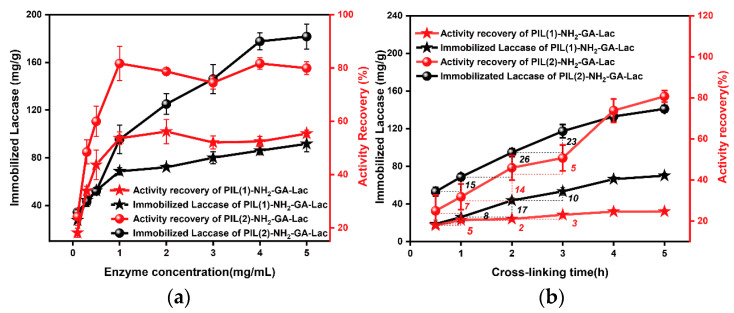
Effect of laccase concentration (**a**) and cross-linking time (**b**) on the activity recovery and the immobilized laccase.

Next, two important immobilized laccase parameters, the initial laccase concentration and the cross-linking time, were investigated. The number of amino-functionalized PIL particles determines the number of laccases. As shown in Figure 3a, with a higher initial concentration, more laccase was able to be immobilized. In addition, the immobilized efficiencies of laccase corresponded to the nature of PILs, such as their specific surface area and pore size. A higher specific surface area and larger pore size can facilitate the entry of enzymes into the support to achieve a higher enzyme immobilization [40]. When the initial concentration of laccase was below 1 mg/mL, both the immobilized laccase and activity recovery increased. It should be noted that when the concentration was 1 mg/mL, the maximum laccase activity recovery was obtained (82%, 0.34 u/mg; the specific activity of free laccase was 0.41 u/mg under the same test conditions). However, the activity recovery did not increase when the concentration was over 1 mg/mL, which might have been due to the fact that an aggregation of the excessive laccase in the pores occurred or that a multilayer structure formed on the surface of the PILs.

Cross-linking time is another parameter that influences the immobilization efficiency of enzymes. As shown in Figure 3b, the activity recovery of the laccase activity increased from 1 to 5 h due to sufficient cross-linking. However, the immobilization rate gradually slowed as the time extended, as shown by the slope (comparing the data in the figure, it was found that the maximum immobilized rate generally occurred within 2 h) in Figure 3b. For example, when the cross-linking time was 2 h, the immobilized laccase of PIL (1)–NH_2_–GA–Lac and PIL (2)–NH_2_–GA–Lac increased by 17 mg/g and 26 mg/g, respectively. In addition, the activity recovery of PIL (2)–NH_2_–GA–Lac increased by 14% during this period. This was the most efficient period of enzyme immobilization. From the viewpoint of dynamics, a large concentration gradient in the early cross-linking time (0.5–2 h) promoted the immobilization of laccase. A maximum activity recovery was achieved when the cross-linking time was around 5 h (i.e., 82%, 0.31 u/mg). It is worth noting that PIL (2)–NH_2_–GA–Lac, having a larger specific surface area, had a higher capture ability for laccase than PIL (1)–NH_2_ under the same enzyme concentration. However, the excessive immobilization of enzymes makes the aperture of the support relatively thin, resulting in the reduced accessibility of the substrate to the active sites [41,42].

### 2.3. pH and Thermal Stability of Immobilized Laccase

**Figure 4:**
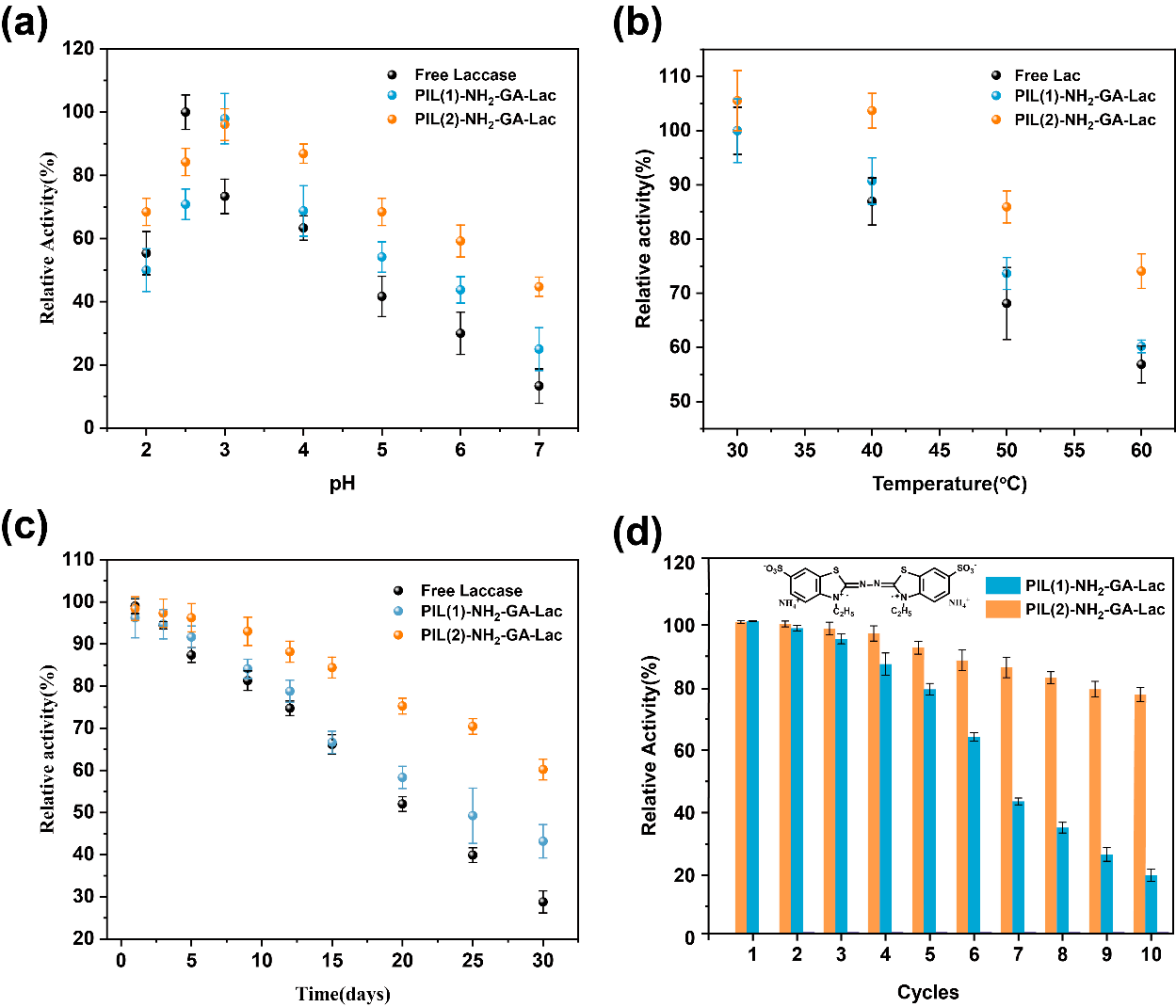
Effect of (**a**) pH and (**b**) temperature on the activity of free laccase and PIL–NH_2_–GA–Lac. (**c**) Storage stability of free and immobilized laccase and (**d**) reusability of the immobilized laccase. Conditions: ABTS (100 mM), PIL–NH_2_–GA–Lac (10 mg/mL), pH = 3 (**b**–**d**), and 25 °C (**a**,**c**,**d**).

The optimal pH of a laccase immobilization reaction system varies according to the charge in the enzymatic protein and the support used [43]. As shown in Figure 4a, and consistent with previous studies, free laccase showed maximum activity at a pH of 2.5 and rapidly decreased activity as the pH increased, and it was almost completely inactivated under neutral conditions [44]. In general, anionic supports attract protons from a given solution, which results in a higher concentration of H^+^ in the diffusion layer of an immobilized enzyme than in the external solution. This is why the pH of the external solution should be higher [45]. In the present study, the amino-functionalized PILs were positively charged (+1.7–+5.8 eV); however, the optimal pH value of the immobilized laccase still was higher than that of free laccase. This can be attributed to the imidazolium skeleton in the PILs being positively charged, and the fact that part of the OH^−^ was enriched in the solution. Therefore, there was more available H^+^ near the laccase at the other end of the cross-linking arm. Further, this is why the optimum pH of the PIL (2)–NH_2_ –GA–Lac system was increased slightly. Additionally, the activity–pH curve of most of the immobilized enzymes was bell-shaped, and compared with that of the free laccase, the bell-shaped curve of the immobilized laccase, especially PIL (2)–NH_2_, was flatter. Even in the buffer solution of pH 7, PIL (2)–NH_2_ was able to retain more than 45% activity.

Figure 4b shows that the relative activities of both the free and immobilized laccase were strongly temperature-dependent. The thermal stability of PIL–NH_2_–GA–Lac was improved, and its activity decreased more slowly than that of free laccase. In particular, PIL (2)–NH_2_–GA–Lac maintained more than 70% activity at 60 °C. This may be attributed to the PIL supports’ providing covalently attached sites and confined space for the laccase, as well as the inactive active center’s being preserved by its binding with the ligand, resulting in the decreased formation of the thermo-unfolded enzyme. Therefore, higher temperatures (above 40 °C) were required to break down the intermolecular interactions and to inactivate the enzyme [46]. In addition, some reports have proved that PILs have a good heat insulation effect, so when the temperature of the external solution rises, the surface and internal temperature of the support will be relatively low, which protects the laccase from being inactivated by high temperatures [47].

### 2.4. Operational Stability of Immobilized Laccase

Storage stability and reusability are also important characteristics and are prerequisites for the practical application of immobilized enzymes. Storage is particularly difficult because the conformation of an enzyme changes over time and gradually loses its activity in solution. As shown in Figure 4c, it can be observed that with the increase in storage time, the difference between the free and immobilized laccase activities became significant. The relative activity of the free laccase was less than 50% after 15 days, while PIL (2)–NH_2_–GA–Lac was still able to maintain more than 80%. After 30 days, the relative activity of PIL (2)–NH_2_–GA–Lac was twice that of free laccase. The storage stability of PIL (1)–NH_2_–GA–Lac fell between that of PIL (2)–NH_2_–GA–Lac and free laccase. This shows that immobilization significantly improved the storage stability of the laccase. One possible reason for this is that the laccase was in a confined space provided by PIL–NH_2_ and that the spatial conformation had little or no tendency to change [48].

The reusability test performed over 10 successive catalytic cycles showed that the immobilized laccase exhibited superior circularity. Despite its loss of activity in cycling, the immobilized laccase (i.e., PIL (2)–NH_2_–GA–Lac) exhibited good reusability, retaining 80% of its initial activity after 10 cycles (Figure 4d). It should be noted that the amount of immobilized laccase in PIL (2)–NH_2_–GA–Lac was about two-fold higher than that in PIL (1)–NH_2_–GA–Lac under theoretical conditions; however, the relative activity of PIL (2)–NH_2_–GA–Lac was three-fold higher than that of PIL (1)–NH_2_–GA–Lac after 10 cycles. This can be attributed to the higher specific surface area and stronger binding force of PIL (2)–NH_2_, by which the laccase activity loss of PIL (2)–NH_2_–GA–Lac was smaller under the same experimental conditions.

### 2.5. Analysis of Enzyme Kinetic Parameters

The enzymatic kinetic parameters are shown in Table 1 and Supplementary Materials: Figure S2. The Michaelis–Menten constant reflects the affinity of an enzyme to a substrate. The smaller the K_m_, the greater the affinity between the enzyme and the substrate. As shown in Table 1, the K_m_ of the immobilized laccase was slightly reduced compared with that of the free laccase, indicating its substrate affinity was enhanced. This might be ascribed to the rough surface of the PILs, which is conducive to interactions with the substrates and enzymes. The maximum reaction rate was V_max_, and K_m_/V_max_ was used to evaluate the catalytic efficiency. The reaction rate of the immobilized enzymes was improved compared with that of free laccase. This may have been due to the enrichment of the substrate on the supports, which made it easier for the laccase to contact the substrate (ABTS) and accelerate the reaction.

### 2.6. Interference Test of Metal Ions

As can be seen from Figure S3, both Fe^3+^ and Cr^2+^ had certain inhibitory effects. This may have been because these metal ions occupy the active centers of laccase. However, Mg^2+^, Cu^2+,^ and Zn^2+^ were able to promote the activity of the laccase. This is consistent with previous results that have found that Zn^2+^ and Mg^2+^ are catalytic activators of many oxidoreductases [49,50]. The activation effect of Cu^2+^ was consistent with the fact that Cu^2+^ is an active center of laccase and an important component of the laccase molecule [51]. Compared with the free laccase, the support provided some shielding effect for the immobilized laccase and reduced the interference of the metal ions.

### 2.7. Removal of 2,4-DCP

**Figure 5:**
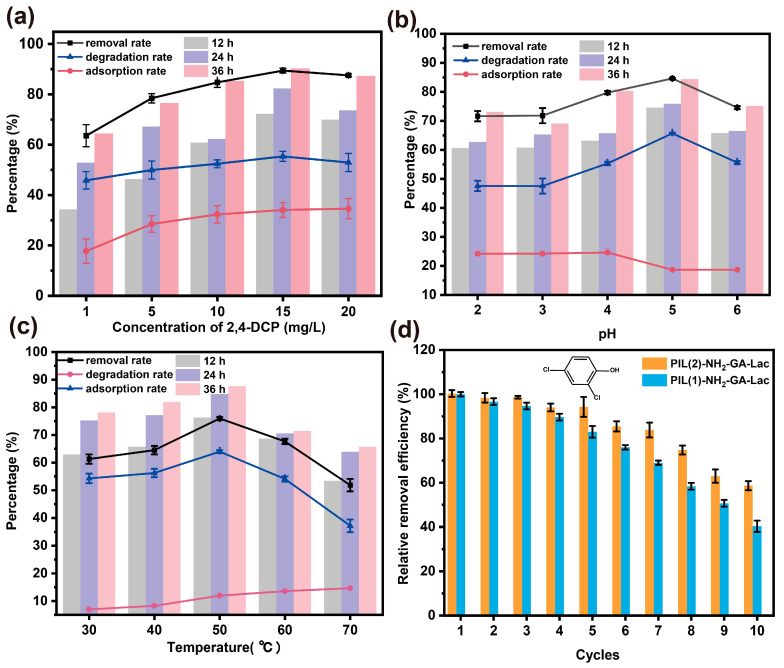
Effect of time and concentration of 2,4-DCP (**a**), pH (**b**), and temperature (**c**) on the efficiency of removal of phenolic compounds and reusability of the immobilized laccase (**d**). Conditions: 2,4-DCP (1 mg/L), PIL–NH_2_–GA–Lac (10 mg/mL), pH = 3 (**b**–**d**), 20 °C (**a**,**c**,**d**), and 12 h (**d**).

Next, the catalytic potential of PIL (2)–NH_2_–GA–Lac was investigated. Many previous studies have shown that various process parameters may significantly affect the removal of harmful pollutants by enzymes [52]. Therefore, after evaluating the stability and reusability of immobilized laccase, it was crucial to determine the effects of various process conditions (e.g., pH, temperature, and substrate concentration) on its efficiency and the optimal conditions for the effective removal of phenolic contaminants. In Figure 5a–c, the bar represents the efficiency of phenol removal over time (12–36 h). The dot plot represents the removal, degradation, and adsorption of 2,4-DCP by the immobilized enzyme within a certain time range. The recycling efficiency of 2,4-DCP removal by the immobilized enzyme was also studied, as shown in Figure 5d.

As shown in Figure 5a, with the increase in the substrate concentration from 1 to 20 mg/L, both the 2,4-DCP removal and degradation increased firstly and then slightly decreased. When the concentration was 15 mg/L, the maximum 2,4-DCP removal and degradation occurred (90% and 55%, respectively). However, the 2,4-DCP removal and degradation decreased slightly when the substrate concentration was 20 mg/L. This can be explained by the fact that at a higher substrate concentration, the phenolic substrate tended to aggregate through intermolecular interactions, which led to a reduction in the substrate adsorption in the immobilized system [53,54]. In addition, it can be seen from the bar that with extension of the reaction time (12–36 h), the removal of 2,4-DCP basically showed a steady rising trend. As one item that contributed to the removal, the 2,4-DCP adsorption increased until it reached around 35%. This also can be understood as the gradual saturation of the immobilized capacity on the support [55].

The removal of 2,4-DCP was investigated over a broad pH range of 2 to 6 at 20 °C (Figure 5b). As can be seen from the bar, when the reaction lasted for 24 h, the 2,4-DCP removal increased substantially, reaching its maximum increment at pH 4. Furthermore, regarding their variation with the pH values, the removal and degradation showed similar trends, while the adsorption did not change significantly, remaining at about 20%. It was also found that the removal changed a little when the pH was 2–4 and reached its maximum value at pH 5 (84%, 36 h). However, when the pH exceeded 5, the degradation was negatively correlated with the pH. It can be seen that the weak acidity of the solution was conducive to the removal and degradation of 2,4-DCP. In a slightly high pH environment, the dissociation equilibrium of 2,4-DCP shifted in the direction of producing more H^+^. According to the principle of chemical reaction equilibrium, anions (OH^−^) enable and promote the catalytic oxidation reaction [56]. However, in this study, this led to lower catalytic oxidation efficiency when the pH value was further increased. On the other hand, the increase in anions in the dissociation equilibrium brought about the formation of free phenoxy, which gave rise to C-O coupling and further led to the accumulation of 2,4-DCP dimers and a high concentration of oligomer. This may hinder the substrate molecules’ ability to enter the laccase’s active center, resulting in reduced 2,4-DCP removal [43].

Figure 5c demonstrates that PIL (2)–NH_2_–GA–Lac was characterized by 2, 4-DCP removal over a wide temperature range. As the temperature increased, the removal and degradation gradually increased, with the maximum removal recorded at 50 °C (24 h, 85%). The covalent binding between the laccase and the PIL–NH_2_ led to the stronger conformation of the laccase, by which the higher temperature was able to make the laccase-activated groups more active, benefiting the diffusion and electron transfer of the substrate in the material channel. In addition, the synergistic mechanism between the laccase and the support enabled the maximum removal of 2,4-DCP by PIL (2)–NH_2_–GA–Lac at 50 °C. However, when the temperature continued to increase, this was not conducive to the adsorption and degradation of 2,4-DCP by PIL (2)–NH_2_–GA–Lac. This was because constant high temperatures will cause protein inactivation and greatly reduce the catalytic oxidation capacity of laccase [57]. The trend of the bar also supports this view. The increase in 2,4-DCP removal decreased with time, reaching its minimum value at 60–70 °C.

The cycling properties of the immobilized laccase system are shown in Figure 5d. As expected, the relative removal efficiency of the immobilized laccase system did not decrease significantly as the number of batches increased. After seven cycles, the 2,4-DCP removal was still maintained at more than 80%. Table S1 (in Supplementary Materials) summarizes recent studies on enzyme immobilization and its application in the degradation of phenolic pollutants. Compared with other supports, PILs have higher mechanical properties, more suitable specific surface area and superior circulation performance. They conform with the support selection principle of enzyme immobilization and provide ideas for the further development of enzyme immobilization. Compared with the typical reports listed, the PIL (2)–NH_2_–GA–Lac system used in this paper showed comparable or better operational stability for the degradation of phenolic pollutants. This can be attributed to the strong binding between the laccase and the support, which helped avoid leakage [58]. The above results illustrate that the reusability of the current immobilized laccase system showed great potential for the system’s large-scale application in the future.

## 3. Experimental Section

### 3.1. Materials

Laccase was supplied by Chengdu Huaxia Chemical (0.5 U mg^−1^). Glutaraldehyde (25% in H_2_O, *v*/*v*) was supplied by Sigma Aldrich (St. Louis, MO, USA). Divinyl benzene (DVB), toluene and 2,2′-azo ratio (2-methylpropanitrile) (AIBN, 98%) were provided by Tianjin Damao Chemical Reagent Co., Ltd. (Tianjin, China). Polyvinyl alcohol purchased from Jiangsu Aikang Biomedical Research and Development Co., Ltd. (PVA). N-vinylimidazole and 3-Bromopropylamine hydrobromide were purchased from Meryer. AIBN was used after fresh recrystallization. 2,2′-Azbis (3-ethylbenzothiazoline-6-sulfonic acid) (ABTS), sodium citrate dehydration, FeCl_3_, AlCl_3_, CrCl_3_, ZnCl_2_, and MgCl_2_ were purchased from Aladdin Chemical Co., Ltd. Except for AIBN, which needs recrystallization, and DVB, which needs extraction, other solvents and reagents were analytical grade and were able to be used without further purification.

### 3.2. Characterization of the Support

The PILs were characterized by scanning electron microscopy (SEM, Haitich SU8020, Tokyo, Japan), Fourier-transform infrared spectroscopy (FTIR, Shimadzu IRTracer-100, Kyoto, Japan), specific surface analysis (BET, BSD-PM, Beijing, China), and thermogravimetric analysis (TGA, Setaram Labsys, Lyon, France). The optical density of the sample solution was measured by a 752 UV-Vis Spectrophotometer (Shanghai Spectral Instrument Co., Ltd., Shanghai, China). The concentration of 2,4-DCP was analyzed by high-performance liquid chromatography (HPLC, Agilent 1260, Santa Clara, CA, USA). The content of the laccase in the solution was determined by a microplate reader (INFINITE M PLEX, Männedorf, Switzerland).

### 3.3. Preparation of Amino PILs

Preparation of amino-ionic liquid monomer (ILM–NH_2_): N-vinylimidazole (2.8 g, 30 mmol) and acetonitrile (30 mL) were added to a two-necked flask equipped with a magnetic stirrer. The mixture was refluxed at 78 °C under a nitrogen atmosphere. 3-Bromopropylamine (4.4 g, 20 mmol) was added drop by drop into the flask over 12 h. After purification, the ILM–NH_2_ was obtained.

Preparation of PIL (1)–NH_2_: ILM–NH_2_ (0.5 g, 2.68 mmol), DVB (3.2 g, 24.6 mmol), ethanol and deionized water were added to a three-necked flask equipped with a magnetic stirrer. The mixture was refluxed at 70 ± 8 °C over 48 h.

Preparation of PIL (2)–NH_2_: Two steps were involved. The first step: according to the traditional suspension polymerization, deionized water (810 g) and PVA (90 g, 5%) were mixed in a three-necked flask equipped with a magnetic stirrer. The mixture was refluxed at 40 °C. Then, toluene and DVB were added (mass ratio: 3:1, 300 g). The mixture was refluxed at 70 °C–80 °C over 2–3 h. The heating was stopped after PDVB solidified. After purification, the PIL (2)–NH_2_ was obtained.

### 3.4. Laccase Immobilization by PILs

Amino-functionalized PILs (named PIL-NH_2_) immobilized laccase (Lac) by covalently binding glutaraldehyde (GA). The activated PIL (1)_–_NH_2_ and PIL (2)_–_NH_2_ (20 mg) were dispersed in a sodium citrate buffer (pH = 3, 10 mL, 100 mM) under ultrasound, and then laccase (1 mg·ml^−1^) was added. Then, glutaraldehyde (2% *v*/*v*) was added drop-wise to the mixture of PIL–NH_2_ and laccase. The new mixture was cross-linked at room temperature for a certain time (e.g., 1, 2, 3, 4, or 5 h). The immobilization efficiency was estimated using Equation (1). Drawing BSA standard curve [59]: Deionized water was used to prepare BSA solution with a concentration of 1–10 μg/mL. BSA (4 mL) was mixed with Coomassie brilliant blue G-250 protein reagent (1 mL), and the absorbance was measured at 595 nm with a microplate reader after 3 min. Coomassie brilliant blue G-250 staining method was used to determine the ability of amino-functionalized PILs to immobilize laccase. After the enzyme was cross-linked with the support for a certain time, the supernatant was taken and combined with the Coomassie brilliant blue G-250 reagent. The absorbance was determined by colorimetry at 595 nm, and C (final concentrations of protein) was obtained according to the standard curve.

(1) $$M=\frac{\left(C_{0}\;-\;C\right)V}{m}$$

Here, M is the efficiency of immobilized laccase of PIL–NH_2_ (mg/g); C_0_ and C are the initial and final concentrations of proteins (μg/mL), respectively; V is the volume of solution (mL); m is the mass of PIL–NH_2_ (mg). The laccase immobilized by PIL–NH_2_–GA–Lac under different initial concentrations of laccase and different cross-linking times was calculated.

### 3.5. Activity Assays of Free and Immobilized Laccase

The activities of the free laccase and immobilized laccase were determined by an ABTS assay at 420 nm. The reaction mixture consisted of 100 mM ABTS and a suitable amount of free or immobilized laccase. During the process, the increase in the absorbance at 420 nm was measured using a UV-2450 spectrophotometer. One unit (U) of laccase activity was defined as the amount of enzyme needed to oxidize 1 mol of ABTS per minute. The specific activity and activity recovery of immobilized enzyme were calculated by the following formulas [60]:

(2) $$\mathrm{The}\;\mathrm{specific}\;\mathrm{activity}\;\mathrm{of}\;\mathrm{the}\;\mathrm{immobilized}\;\mathrm{enzyme}(\frac{\mathrm{IU}}{g})=\frac{△A}{M\;\times \;m\;\times \;t}$$

where ΔA is the change in absorbance in a certain period; M is immobilized laccase (mg/g); m is the mass of PIL–NH_2_ (mg); t is the reaction time (min).

(3) $$\mathrm{Activity}\;\mathrm{recovery}=\frac{R_{i}}{R_{f}}$$

where R_i_ is the specific activity of the immobilized laccase (U/g); R_f_ is the specific activity of the free laccase under the same conditions (U/g).

### 3.6. Effect of pH and Temperature on Laccase Activity

To determine the pH and temperature activity profiles of the free and immobilized laccase, the activity assays were carried out over a pH range of 2.0–7.0 (in sodium citrate buffer (100 mM, pH 2.0–4.0), sodium acetate buffer (100 mM, pH 5.0) and phosphate buffer (100 mM, pH 6.0–7.0) at 25 °C) and a temperature range of 30–60 °C. The results were converted to relative activities (percentage of the maximum activity obtained in that series). Each set of experiments was performed in triplicate, and the arithmetic mean values were calculated.

### 3.7. Storage Stability and Cycle Stability

The free and immobilized laccase were incubated in sodium citrate buffer at 4 °C. To detect the activity, the laccase was taken out of the buffer every few days and tested by ABST assay. The relative activity of freshly immobilized laccase was considered to be 100%.

### 3.8. Determination of K_m_ and V_max_

Determination of K_m_ and V_max_: The free and immobilized laccase were assayed at different concentrations of ABTS, ranging from 1 to 5 mM, to determine the laccase kinetics, i.e., K_m_ and V_max_ values, using Lineweaver–Burk reciprocal plot transformation of Michaelis–Menton equation.

### 3.9. Effect of Metal Ions

Aqueous solutions of Cr^2+^, Fe^3+^, Zn^2+^, Mg^2+^, and A1^3+^ ions (e.g., 5 mmol/L) were prepared. Metal salt solution (1 mL), sodium citrate buffer (1 mL, pH = 3.0), free laccase (1 mL) or immobilized laccase (5 mg) and ABTS substrate (2 mL) were mixed in a 50 mL centrifuge tube. The final concentration of metal ions was 1 mmol/L. Reaction took place at 25 °C for 5 min. The laccase activity was measured at 420 nm wavelength by ultraviolet spectrophotometer. Three parallel samples were set in each group. The buffer solution was used instead of the metal salt solution in the blank group.

(4) $$\omega =\frac{L_{j}}{L_{0}}\times 100\%$$

where *ω* is the metal ions‘ effect on efficiency; *L_j_* is the laccase activity with metal ions; *L*_0_ is the laccase activity without metal ions.

### 3.10. Removal of Phenolic Compounds

The ability of the immobilized laccase to remove 2,4-DCP (1–20 mg/L) with different pH (2–6) and temperature (30 °C–70 °C) levels was tested in 10 mL reaction medium (10 mg PIL (2)–NH_2_–GA–Lac, 12–36 h). Chlorophenols removed by adsorption were also investigated using inactivated PIL–NH_2_–GA–Lac. The conditions were the same as above, except that PIL–NH_2_–GA–Lac was inactivated. Each set of experiments was performed in triplicate, and the arithmetic mean values were calculated.

The removal (*R*), adsorption (*R_A_*) and degradation (*R_D_*) of 2,4-DCP were calculated by the following Equations:

(5) $$R=\frac{C_{1}-C_{2}}{C_{1}}\times 100\%$$

(6) $$R_{A}=\frac{C_{1}-C_{3}}{C_{1}}\times 100\%$$

(7) $$R_{D}=\left(R-R_{A}\right)$$

where *C*_0_ is the initial concentration of 2,4-DCP, *C*_1_ is the residual concentration of 2,4-DCP removed by immobilized laccase, and *C*_2_ is the residual concentration of 2,4-DCP adsorbed by inactivated PIL–NH_2_–GA–Lac.

To explore the recyclability of immobilized enzyme, the degradation of 2,4-DCP (1 mg/L) by PIL (2)–NH_2_–GA–Lac (10 mg) was repeated in 10 cycles. After each batch, the immobilized laccase was isolated by centrifuge, and then an aliquot of fresh reaction medium was added for the next cycle. The relative removal efficiency was correlated with the highest removal percentage (100% represented the highest removal). This was expressed as the following Equation:

(8) $$\mathrm{Relative}\;\mathrm{removal}\;\mathrm{efficiency}=\frac{R_{i}}{R_{max}}\times 100$$

where *R_i_* is the phenol removal of each sample and *R_max_* is the highest phenol removal of all samples. In the cycle test, the first phenol removal was generally considered 100%.

The high-performance liquid chromatography: C18 column. The mobile phase was 75% (mass fraction) methanol and 25% (mass fraction) ultrapure water. The flow rate was 1 mL/min. The detection wavelength was 225 nm. The peak time of 2,4-DCP was about 6.5 min at 35 °C.

## 4. Conclusions

In summary, in this study, confined polymerization was demonstrated to be an efficient strategy for constructing PILs with a high specific surface area (302 m^2^/g) and mesoporous structure (~12 nm). (I) The microenvironment provided by PILs may play an important role in shielding external environmental interferences (e.g., pH, temperature). (II) Compared with the free laccase, the immobilized laccase showed improved stability and circulation stability. (III) In the catalytic study of the effects of the immobilized laccase on 2,4-DCP, the interactions between the PILs and 2,4-DCP might have enriched the substrates, enabling the laccase to promote biodegradation. This research demonstrated the versatile potential of PILs in constructing high-efficiency immobilized enzymes, and lends support to the promotion of their application in wastewater treatment and biological technology development.

## Figures and Tables

**Table 1:** K_m_ and V_max_ of free laccase and immobilized laccase.

	V_max_ (mM/(mg·min))	K_m_ (mmol/L)
Free laccase	0.035	1.202
PIL (1)–NH_2_–GA–Lac	0.043	1.100
PIL (2)–NH_2_–GA–Lac	0.043	0.998

## Data Availability

Not applicable.

## Supplementary Materials

The following supporting information can be downloaded at: https://www.mdpi.com/article/10.3390/molecules28062569/s1, Figure S1: The H1 NMR of ionic liquids monomer(ILM, [AVIM]Br); Figure S2: Linewaever-Burk plots of free laccase and immobilized laccase; Figure S3: Effect of metal ions on laccase activity. Table S1: Different support enzyme loading performance and phenol compounds removal rate.
