# Supplementary material for: Mesoporous Polymeric Ionic Liquid via Confined Polymerization for Laccase Immobilization towards Efficient Degradation of Phenolic Pollutants
Source: Molecules. 2023 Mar 12;28(6):2569. doi: 10.3390/molecules28062569 (PMC10059984; doi:10.3390/molecules28062569)
Supplement: Supplementary file 1 [file molecules-28-02569-s001.zip › molecules-2248683-supplementary.pdf]

Supplementary data for:

Mesoporous Polymeric Ionic Liquid via Confined Polymerization for Laccase Immobilization towards Efficient Degradation of Phenolic Pollutants

Yu Liang [1], Xinyan Chen [1], Jianli Zeng [2], Junqing Ye [1], Bin He [1], Wenjin Li [1] and

and Jian Sun [1,3,*]

[1] Key Laboratory of Molecular Medicine and Biotherapy in the Ministry of Industry and Information Technology, School of Life Science, Beijing Institute of Technology, Beijing 100081, China

[2] State Key Laboratory of Catalytic Materials and Reaction Engineering, Research Institute of Petroleum Processing, SINOPEC, Beijing 10083, China

[3] Advanced Research Institute of Multidisciplinary Science, Beijing Institute of Technology, Beijing 100081, China

[*]Corresponding author email address: jiansun@bit.edu.cn

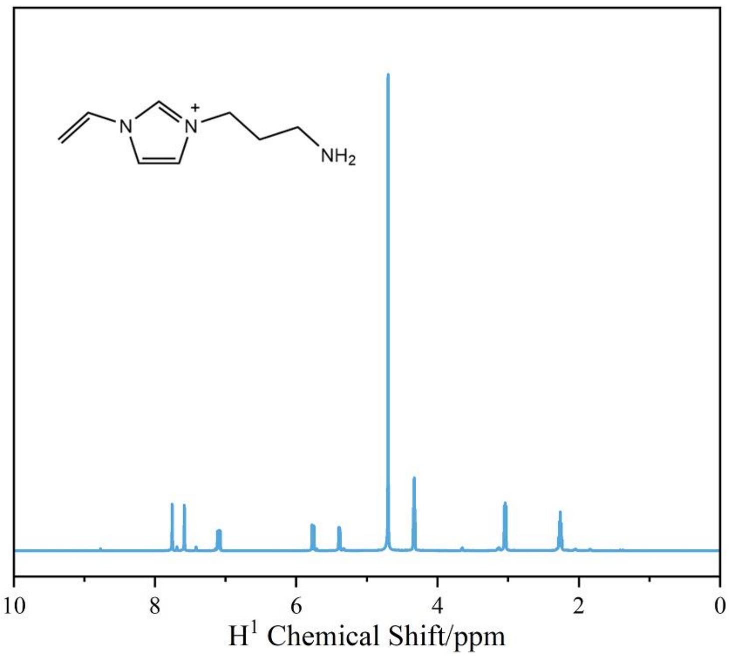

Figure S1. The H1 NMR of ionic liquids monomer(ILM, [AVIM]Br).

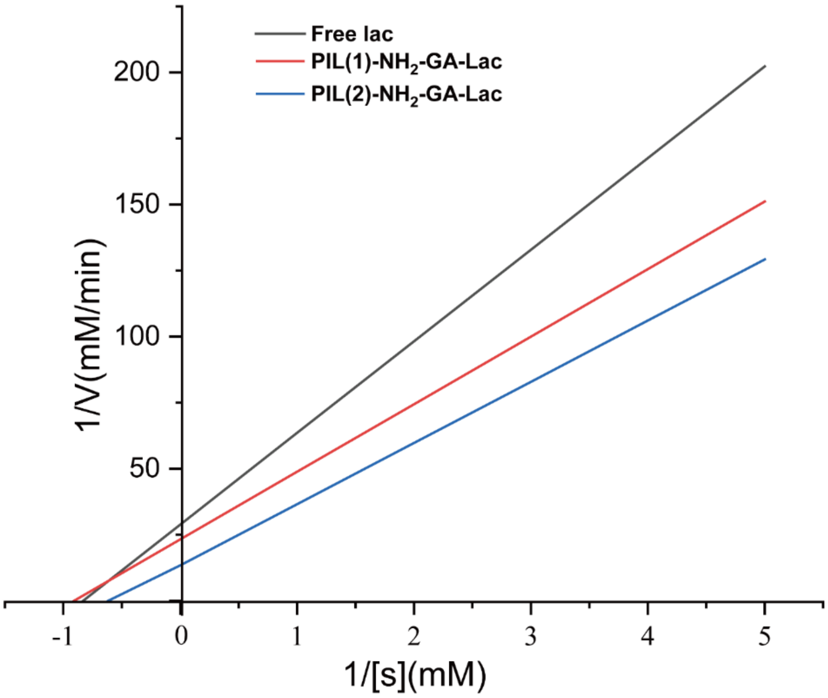

Figure S2. Linewaever-Burk plots of free laccase and immobilized laccase.

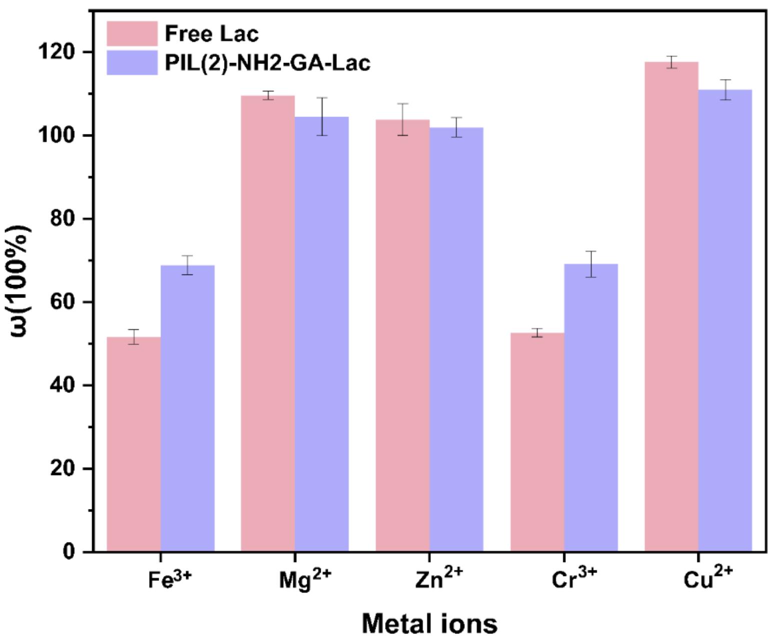

Figure S3. Effect of metal ions on laccase activity.

Table S1. Different support enzyme loading performance and phenol compounds removal rate.

Carrier	Enzyme loading (mg/g)	Storage stability (days, Relative activity)	Cycling stability (Times, Relative activity)	Phenolic compound, removal	Ref.
Cu (II)-chelated chitosan nanoparticles	66	/	8, 50%	Phenol, 87%	[60]
chitosan–clay	75%	42 d, 55%	10, 75%	Phenol, 80%	[24]
Silica	30	/	5, 61%	Catechol, 95%	[13]
Chitosan/poly (vinyl alcohol)	853	10 d, 60%	7, 54%	2,4-DCP, 87.6%	[21]
magnetic nanoparticles	85.8%	30 d, ~80%	6, 83%	Phenol, 86.1%	[36]
PIL-NH_2	181	15 d, 84%	10, ~80%	2,4-DCP, 90%	This work
